# A Man with Sore Throat—A Case Report

**DOI:** 10.21980/J8MH0B

**Published:** 2023-04-30

**Authors:** Nathan Mercado, Sawyer Schuljak, Daniel Ng, Curtis Knight, Allison Woodall, John Costumbrado

**Affiliations:** * University of California, Riverside School of Medicine, Riverside, CA; ^ Riverside Community Hospital / University of California Riverside, Department of Emergency Medicine, Riverside, CA

## Abstract

Supraglottic thermal burns resulting from ingestion of excessively hot food or drink can potentially lead to fatal airway obstruction due to severe edema. In this case we evaluate an adult male who presented to the emergency department (ED) with sore throat and mild voice hoarseness that began while eating hot rice soup two days prior. The patient states that after taking a bite of the hot soup, he coughed due to the heat. Shortly after, he felt a burning sensation in his throat and developed a foreign body sensation. A visual examination with video laryngoscopy of the upper airway showed no evidence of foreign bodies; however, there were suspected thermal burns near the patient’s epiglottis. This case demonstrates how thermal burns can be evaluated and treated with conservative measures to reduce edema, but care takers should be aware of the severe burns leading to complete airway obstruction.

**Topics:**

Supraglottic burns, airway obstruction, laryngoscopy.

**Figure f1-jetem-8-2-v16:**
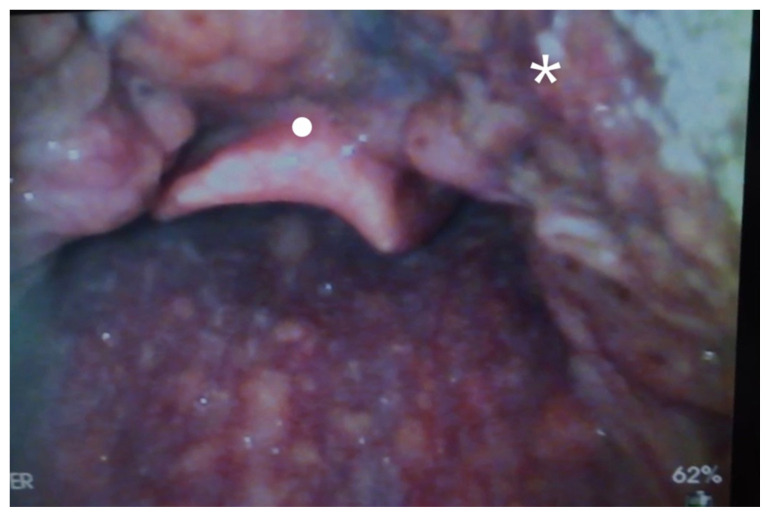


**Figure f2-jetem-8-2-v16:**
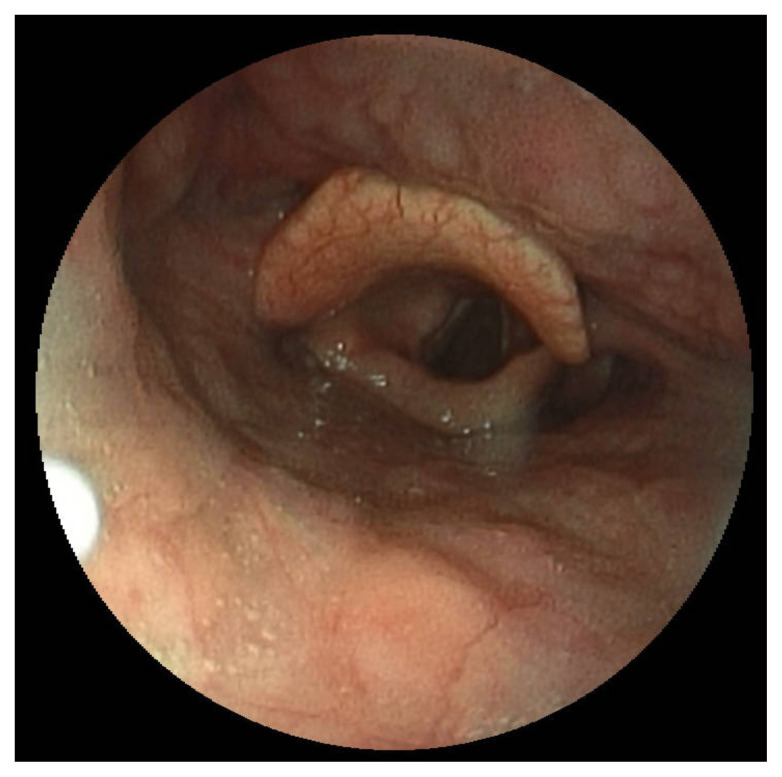


**Figure f3-jetem-8-2-v16:**
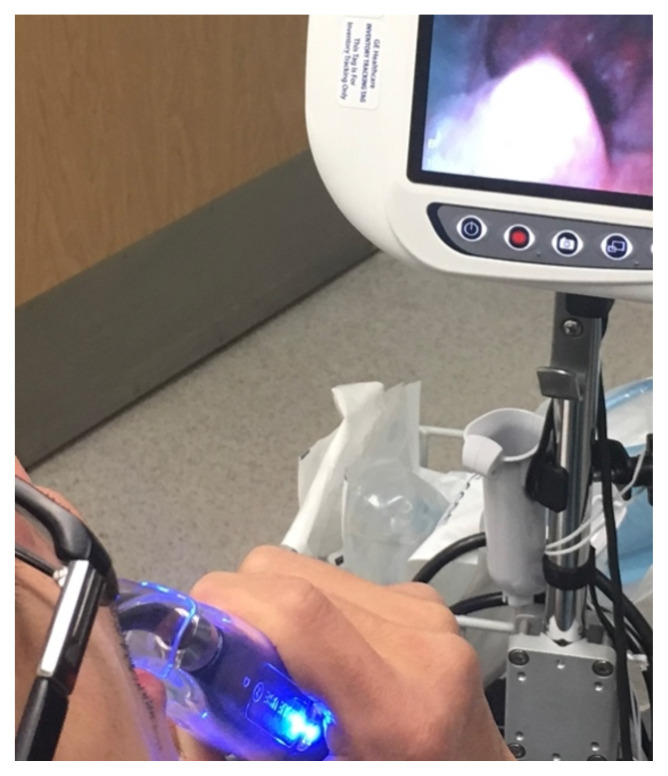


## Brief introduction

[Fig f1-jetem-8-2-v16][Fig f2-jetem-8-2-v16][Fig f3-jetem-8-2-v16]The direct application of heat to the epiglottis and surrounding tissue by hot food, liquid, or vapors can lead to acute airway injuries such as thermal epiglottitis that increase the risk for acute airway obstruction.^1^ Thermal burns of the oral cavity and pharynx are common among infants and children; however, they can rarely occur in adults.^2^–^5^ While serious and life threatening thermal injuries are uncommon amongst adults, most can be successfully treated with supportive care.^6^ Although fatal cases are rare, there should be a high clinical concern for the possible development of airway obstruction due to severe edema and inflammation when evaluating patients with thermal injuries.

## Presenting concerns and clinical findings

A 30-year-old male presents to the ED with sore throat and mild voice hoarseness that started while eating hot rice soup two days prior. He coughed while eating and shortly thereafter developed a foreign body sensation in his throat. Risks, benefits, and alternatives including sedation, airway topicalization with lidocaine, and fiberoptic evaluation were discussed, and the patient agreed to the team performing a modified Tomahawk technique to visualize the airway without sedation or topicalization. Written consent was obtained for use of the patient’s images for this case report.

## Significant findings

Video laryngoscopy of the upper airway was performed two days after initial burn injury. The images obtained demonstrated laryngeal edema and inflammation near the epiglottis. The dot identifies the epiglottis and the asterix identifies the area of moderate thermal burns. Imaging also demonstrated adequate patency of airway and ruled out the need for intubation at that time. Image of normal airway with video laryngoscope is provided for comparison.^7^

Of note, the Tomahawk technique is typically used to intubate an awake patient and is performed face-to-face with the patient in an upright, seated position.^8^ Image of the patient performing the modified-Tomahawk technique is shown. Similar to the technique used to drain peritonsillar abscesses, the patient was given the video laryngoscope blade and instructed to depress the base of his tongue and advance as tolerated while the provider adjusted the blade as needed to improve visualization. In this case there was no need to intubate as our patient was protecting his airway; however, this technique allowed inspection of the oropharynx and airway.

## Patient course

During the ED visit, the patient's vital signs were within normal limits. The patient was well-appearing and able to speak and swallow comfortably. He had no stridor or increased work of breathing. Analgesic medication was offered, but the patient declined given the minimal pain. The patient tolerated the visual laryngoscope examination well without complications. Advancement of the laryngoscope was limited due to the lack of topicalization or sedation; however, the patient was instructed to immediately signal if there was discomfort. The patient was given control of the video laryngoscope blade using the modified Tomahawk technique for the patient’s comfort and to avoid eliciting the patient's gag reflex. Given the findings showing moderate thermal burns, the possibility of further observation in the hospital was discussed. However, given the patient’s delayed presentation after the initial injury and overall stable condition, the patient preferred conservative management with close outpatient monitoring. When following up with the patient over the phone, the patient was doing much better. The mild pain in the patient’s throat was well-controlled with oral acetaminophen and ibuprofen. The patient reported that the pain resolved by day four and the foreign body sensation resolved by day five. There were no further complications reported.

## Discussion

Laryngopharyngeal and epiglottic thermal burns secondary to hot food or drink ingestion are rare in adults and usually not severe. The average adult has the insight and protective reflexes such as coughing and spitting to protect against consuming food and drink that are too high in temperature. Therefore, many cases related to intraoral and laryngopharyngeal thermal burns are usually infants or adults with severe mental illness.^9^ Although severe cases are rare, the development of airway obstruction due to severe edema and inflammation should be considered. In this case, soft tofu soup is a Korean cuisine that is served at boiling temperatures. The soft stew may also contain vegetables, meat, and rice, which contributed to the suspicion of a possible foreign body. For those not familiar with such dishes, extra caution must be made when ingesting these types of meals.

Thermal injuries associated with inhalation can range from mild to severe. Mild airway burns may present with cough and sore throat and typically resolve without intervention. Moderate burns may present with increased cough, wheezing, dyspnea, and stridor, and possibly accompanied by erythema and edema of the airway mucosa. These burns generally have a stable clinical course; however, supportive care, monitoring, and possible airway management may be indicated. In contrast, patients with severe airway burns may present with respiratory distress, often due to airway obstruction. Clinical findings may include full-thickness mucosal burns and severe airway edema. Severe burns can be immediately life-threatening due to the high risk of obstruction and respiratory failure, making airway management essential in such cases.^6^

Upper airway swelling may be delayed and not peak until 24 hours after initial injury. Therefore, frequent re-evaluations are necessary to identify the development of an airway obstruction.^6^ Although there is a paucity of guidelines specifically related to the management of supraglottic burns from hot foods and liquids, management is analogous to that of inhalation injuries. The International Society for Burn Management (ISBM) has provided recommendations for airway management and treatment for similar burns. In suspected burns, airway obstruction should be a main concern and endotracheal intubation should be performed if the airway becomes compromised. The patient should be placed in an upright position to improve lymphatic drainage and reduce edema while laryngoscopy can confirm the diagnosis of laryngeal edema warranting further intervention.^10^ However, in less severe cases, the treatment of laryngopharyngeal burns is generally supportive. This includes head elevation, avoidance of further thermal injury, and prevention of further injury from gastroesophageal reflux disease with proton pump inhibitors and H2 receptor antagonists.^9^ Airway edema generally resolves in 3–6 days depending on the severity of injury, and treatment should be individualized for the needs of the patient. The routine use of corticosteroids does not appear to provide benefit.^11^

Airway obstruction should be suspected in patients with recent supraglottic burns due to accidental consumption of food or drink at excessive temperatures. Patients with mild to moderate burns often present with cough, foreign body sensation, and minimal dyspnea and stridor. These patients can typically be managed supportively without the need for airway intervention. In severe cases, such as those presenting with stridor and respiratory distress, laryngoscopy is the preferred imaging modality to identify excessive airway edema and inflammation. A modified Tomahawk technique as described in this case may be useful in assessing patients but preferably with airway topicalization. Timing of thermal injuries also may impact management and observation plans. Inflammation and obstruction associated with thermal injury may not be present until later in the clinical course, thereby necessitating ongoing monitoring of the airway for at least 24 hours until swelling begins to subside.

## Supplementary Information








