# Supplementary figures and images for: A Man with Sore Throat—A Case Report
Source: J Educ Teach Emerg Med. 2023 Apr 30;8(2):V16–9. doi: 10.21980/J8MH0B (PMC10332678; doi:10.21980/J8MH0B)

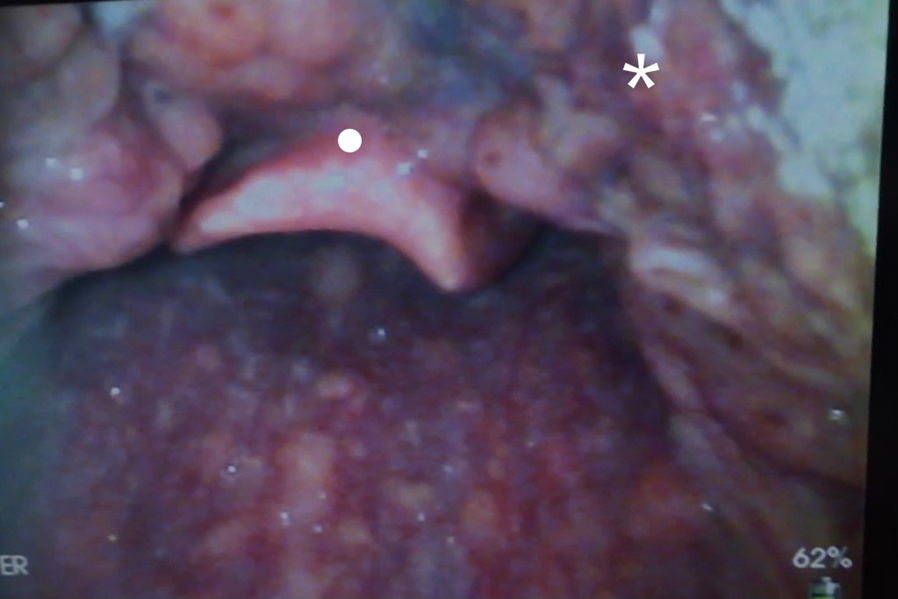

Supplement: Supplementary file 1 [file JETem-8-2-V16-supp1.jpeg]

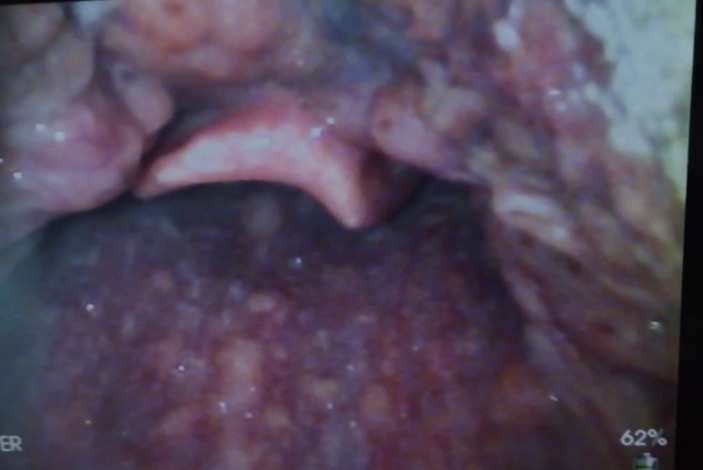

Supplement: Supplementary file 2 [file JETem-8-2-V16-supp2.jpeg]

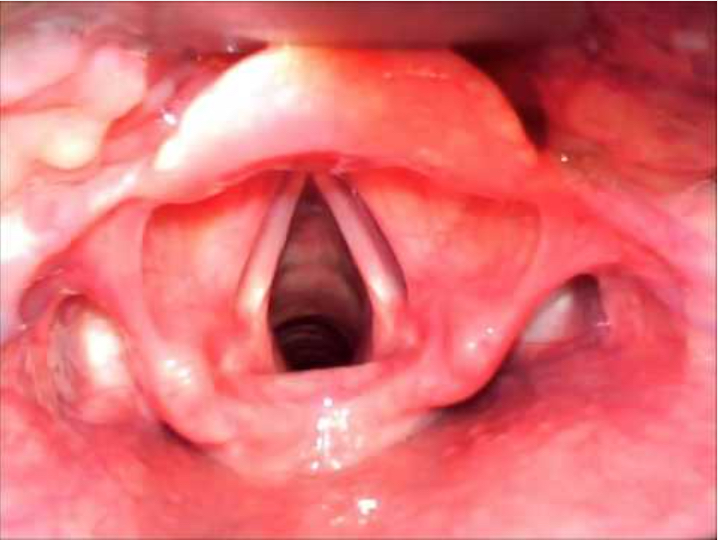

Supplement: Supplementary file 3 [file JETem-8-2-V16-supp3.jpeg]

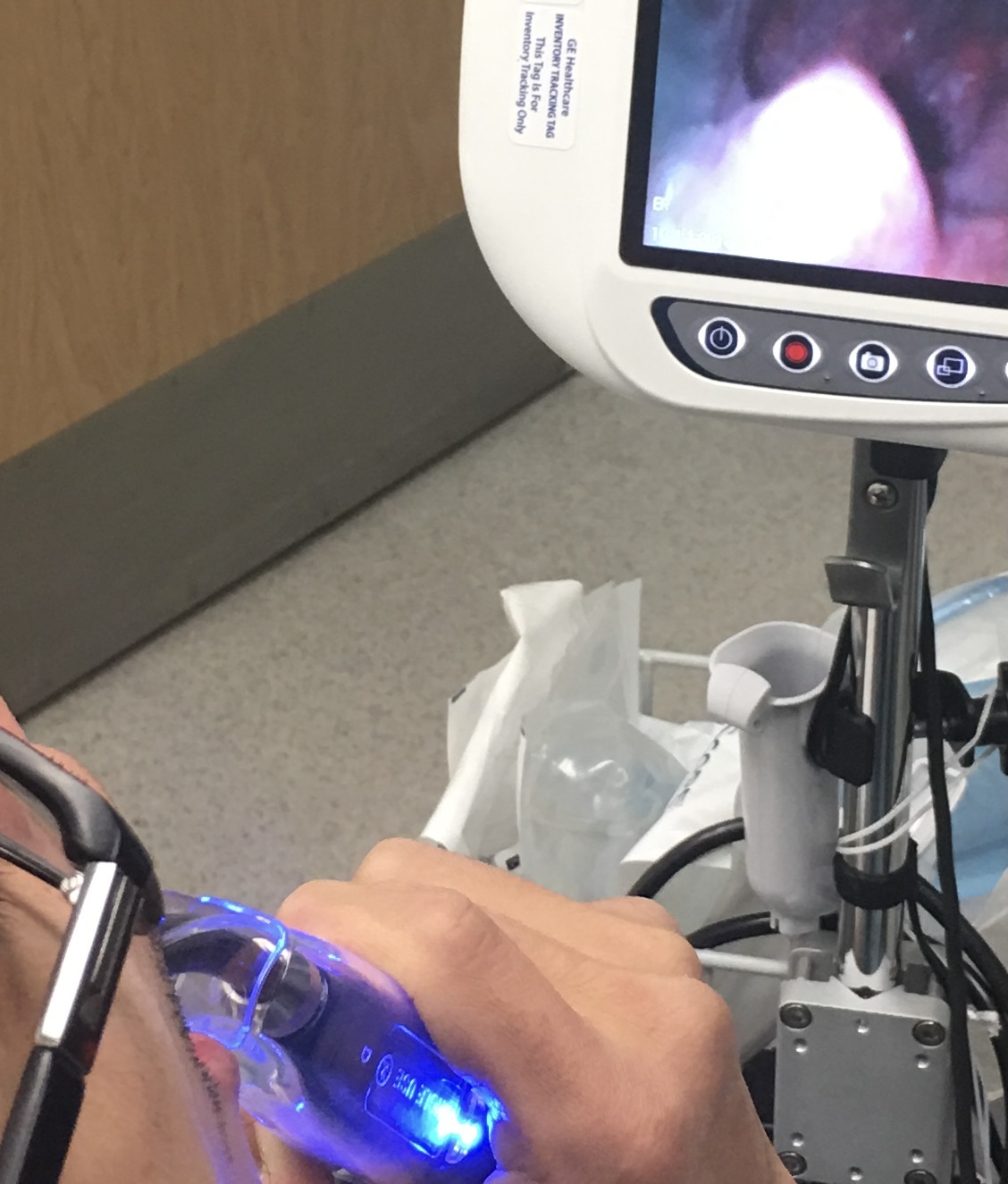

Supplement: Supplementary file 4 [file JETem-8-2-V16-supp4.jpeg]
